# Utilization of Electronic Medical Records and Biomedical Literature to Support the Diagnosis of Rare Diseases Using Data Fusion and Collaborative Filtering Approaches

**DOI:** 10.2196/11301

**Published:** 2018-10-10

**Authors:** Feichen Shen, Sijia Liu, Yanshan Wang, Andrew Wen, Liwei Wang, Hongfang Liu

**Affiliations:** 1 Department of Health Sciences Research Mayo Clinic Rochester, MN United States

**Keywords:** electronic medical record, literature, text mining, rare diseases

## Abstract

**Background:**

In the United States, a rare disease is characterized as the one affecting no more than 200,000 patients at a certain period. Patients suffering from rare diseases are often either misdiagnosed or left undiagnosed, possibly due to insufficient knowledge or experience with the rare disease on the part of clinical practitioners. With an exponentially growing volume of electronically accessible medical data, a large volume of information on thousands of rare diseases and their potentially associated diagnostic information is buried in electronic medical records (EMRs) and medical literature.

**Objective:**

This study aimed to leverage information contained in heterogeneous datasets to assist rare disease diagnosis. Phenotypic information of patients existed in EMRs and biomedical literature could be fully leveraged to speed up diagnosis of diseases.

**Methods:**

In our previous work, we advanced the use of a collaborative filtering recommendation system to support rare disease diagnostic decision making based on phenotypes derived solely from EMR data. However, the influence of using heterogeneous data with collaborative filtering was not discussed, which is an essential problem while facing large volumes of data from various resources. In this study, to further investigate the performance of collaborative filtering on heterogeneous datasets, we studied EMR data generated at Mayo Clinic as well as published article abstracts retrieved from the Semantic MEDLINE Database. Specifically, in this study, we designed different data fusion strategies from heterogeneous resources and integrated them with the collaborative filtering model.

**Results:**

We evaluated performance of the proposed system using characterizations derived from various combinations of EMR data and literature, as well as with sole EMR data. We extracted nearly 13 million EMRs from the patient cohort generated between 2010 and 2015 at Mayo Clinic and retrieved all article abstracts from the semistructured Semantic MEDLINE Database that were published till the end of 2016. We applied a collaborative filtering model and compared the performance generated by different metrics. Log likelihood ratio similarity combined with k-nearest neighbor on heterogeneous datasets showed the optimal performance in patient recommendation with area under the precision-recall curve (PRAUC) 0.475 (string match), 0.511 (systematized nomenclature of medicine [SNOMED] match), and 0.752 (Genetic and Rare Diseases Information Center [GARD] match). Log likelihood ratio similarity also performed the best with mean average precision 0.465 (string match), 0.5 (SNOMED match), and 0.749 (GARD match). Performance of rare disease prediction was also demonstrated by using the optimal algorithm. Macro-average *F*-measure for string, SNOMED, and GARD match were 0.32, 0.42, and 0.63, respectively.

**Conclusions:**

This study demonstrated potential utilization of heterogeneous datasets in a collaborative filtering model to support rare disease diagnosis. In addition to phenotypic-based analysis, in the future, we plan to further resolve the heterogeneity issue and reduce miscommunication between EMR and literature by mining genotypic information to establish a comprehensive disease-phenotype-gene network for rare disease diagnosis.

## Introduction

### Background

In the United States, a rare disease is described as the one affecting no more than 200,000 patients at a certain time [1]. Currently, there are nearly 10% Americans suffering from rare diseases [2]. However, patients often are misdiagnosed or left undiagnosed because of insufficient clinical knowledge and experience. Furthermore, merely 5% of these diseases have treatment plans [2]. Therefore, accelerating rare disease diagnostic decision support is crucial and urgent.

The very initial step in diagnosing rare disease is to stratify patients into subgroups with similar phenotypic characterizations. In addition, with computationally accessible medical data growing at an exponential rate, an abundance of rare disease-related phenotypic information is believed to be buried in electronic medical records (EMRs) and medical literature. Therefore, we hypothesize that patients’ phenotypic information available among these resources can be leveraged to accelerate disease diagnosis. Few studies focus on phenotypic characterization of diseases and the analysis of phenotype-disease associations from free-text data such as EMRs and medical literature. One of the most representative efforts, the Human Phenotype Ontology (HPO) [3] was built to collect human phenotypic information for the differential diagnosis of rare diseases. In our previous work, we leveraged the HPO to annotate a large collection of clinical narratives and demonstrated a use case by using an annotation pipeline to perform knowledge discovery on Wilson disease [4]. We also proposed the use of collaborative filtering in our previous study for rare disease diagnosis [5], as making diagnostic decision making for a patient based on phenotype is similar to recommending a similar online product according to customers’ previous purchases in e-commerce [6-8].

Since all datasets are flawed, it is important to prepare data with good quality, as machine learning depends heavily on data [9]. Especially for collaborative filtering algorithm, a proper preparation of data can largely avoid key information loss and improve learning performance [10]. More challenges come into the picture while feeding heterogeneous data into collaborative filtering model.

### Previous Work

One of these challenges is the alignment of semantic heterogeneity. Semantic heterogeneity is referred to as a situation where 2 or more datasets are provided by different parties with various perspectives and purposes [11]. For structured data, the fusion of heterogeneous data is difficult due to inconsistent data models, data schemas, query languages, and terminology [12]. For unstructured or semistructured data, such issues are exacerbated as schemas must become much more flexible to accommodate the nonstandardized data and as such semantic drift becomes a more significant problem. Some studies have focused on making good semantic alignment across heterogeneous data. For example, MedKDD is a system for integrating and aligning heterogeneous biomedical ontologies [13]. Bache et al targeted on identifying patient cohort from heterogeneous resources by developing an adaptive query model [14]. Bleich et al made a comparison between integrated and interfaced hospital systems [15]. Burkle et al conducted a study to transfer data stored in one electronic patient record to another health care information system [16]. EHR4CR demonstrates an interoperable way to reuse electronic health records [17]. Mate et al conducted a study on integrating ontology data between clinical and research systems [18]. SHRINE provides a platform for disease studies across multiple health care institutions [19]. Ohmann et al proposed an overview of studies on data interoperability of basic research, clinical research, and medical data [20].

Another challenge is to get benefit from heterogeneous data to improve performance of machine learning. To investigate this, Lewis et al applied support vector machine on heterogeneous biological data to infer gene function [21]. Yu et al introduced a l2-norm multiple kernel learning algorithm and applied it on biomedical data fusion [22]. Ye et al showed a study on Alzheimer disease using heterogeneous data fusion [23]. Wang et al made a comparison among clinical notes, biomedical literature, and their combination to test their performances with word embeddings [24]. Torii et al showed the performance for concept extraction using machine learning taggers across narratives from heterogeneous data sources [25]. A GOstruct extension was developed to annotate protein functions from heterogeneous data [26].

### Objective

According to the aforementioned related work, although some success was demonstrated, the issue regarding semantic heterogeneity is still an unsolved puzzle. Moreover, to the best of our knowledge, no study has paid attention to the impact of applying collaborative filtering on heterogeneous data, especially in biomedical domain. Therefore, it is interesting to investigate how data fusion strategies on heterogeneous resources can work with collaborative filtering for an optimal recommendation.

In this work, we developed a new framework based on our previous designed collaborative filtering system to incorporate heterogeneous data sources with different data fusion strategies to assist in diagnosing rare diseases. We extracted Unified Medical Language System concepts with MetaMap [27] and applied the HPO with the Genetic and Rare Diseases Information Center (GARD) [28] to annotate clinical notes at the Mayo Clinic generated from 2010 to 2015 as well as research articles stored in the Semantic MEDLINE Database (SemMedDB) [29] published up to December 2016. We integrated different data fusion strategies with collaborative filtering and evaluated their performances for patient recommendation and rare disease prediction.

## Methods

### Data Collection

For the EMR dataset, we collected clinical notes generated at the Mayo Clinic from 2010 to 2015. The extracted corpus maintained about 13 million unstructured clinical notes for over 700,000 patients. We only annotated sections with problems and diagnoses. For the medical literature dataset, we extracted abstracts of research articles from the SemMedDB. We then used HPO and GARD terms to match either subject or object for each predication [29] and finally came up with 91,680 phenotype-rare disease associations to process.

### Collaborative Filtering Model for Rare Disease Recommendation

In e-commerce, collaborative filtering techniques [30] are popularly applied to recommend products to a customer based on customers with similar purchase preferences and other interests. Diagnosing a patient with a disease based on patients’ phenotypic information is very similar to recommending a product to a customer; therefore, it is natural to propose the use of collaborative filtering for disease diagnosis.

In our previous work, we developed a collaborative filtering model based on a cohort of rare disease patients to stratify patients into subgroups and accelerate the diagnosis of rare diseases. Here, we treated patient profiles with their respective phenotypes as binary inputs, which means that the patient either has or does not have a phenotype. For the patients with a confirmed rare disease diagnosis, we used their phenotypes as input and treated their rare disease diagnosis as labels to train the collaborative filtering model.

Specifically, we applied the Tanimoto coefficient similarity (TANI), overlap coefficient similarity (OL), Fager & McGowan coefficient similarity (FMG), and log likelihood ratio similarity (LL) as 4 measurements to compute patient similarity [5]. For any 2 patients m and n, |P_m_| and |P_n_| denote the number of phenotypes each patient has, and TANI, OL, FMG, and LL are described as shown in Equations 1, 2, 3, and 4, respectively.

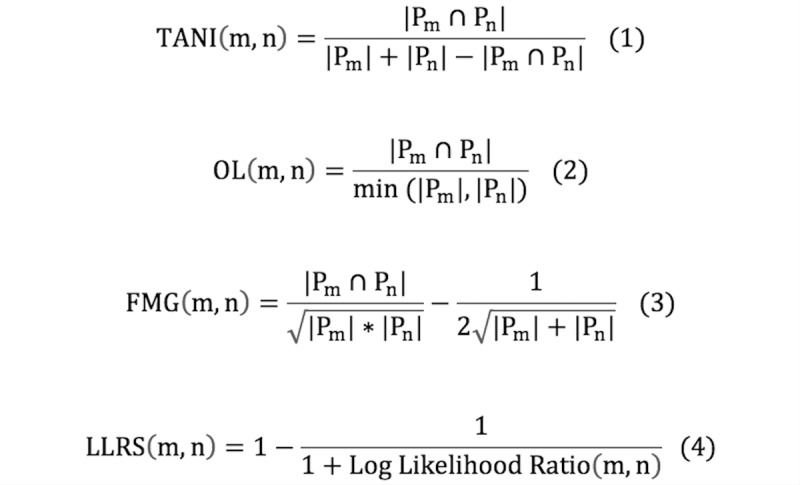



We also applied 2 neighborhood algorithms to provide recommendations: k-nearest neighbors (KNN) and threshold patient neighbor (TPN) [5]. Detailed steps of identifying neighborhood for 2 approaches are shown in Textbox 1.

### Semantic MEDLINE Database

SemMedDB is a repository of semantic predications (ie, subject-predicate-object triples) extracted from the titles and abstracts of all PubMed citations [29,31-33]. In this study, we used SemMedDB Version 25, which contains more than 84 million predications (ie, associations) between concepts retrieved from abstracts of over 25 million PubMed-indexed publications [34].

### Human Phenotype Ontology

The HPO is a standardized vocabulary for phenotypic terms, and it is built based on collecting phenotypic knowledge from various biomedical literature as well as databases. In this study, we used HPO released in September 2016 to annotate phenotypic terms.

### Genetic and Rare Diseases Information Center

The GARD is a database that contains information on rare diseases. It groups collected 4560 diseases into 32 disease categories. In this study, we used the GARD to extract rare disease terms.

**Algorithm 1-Neighborhood identification.**

Input: Sorted Similarity Score Map S (Neighbor_Patient, Score) for each patient, number of neighbor k, similarity threshold t

Output for KNN: Neighbor List LK

Output for TPN: Neighbor List LT

1. FOR each neighbor_patient NP in S

2.      score_np_=S.get(NP)

3.      IF (LK.size()<k)

4.         add NP into LK

5.      IF (score_np_>t)

6.         add NP into LT

7. RETURN LK, LT

### Learning Methods

**Figure 1:**
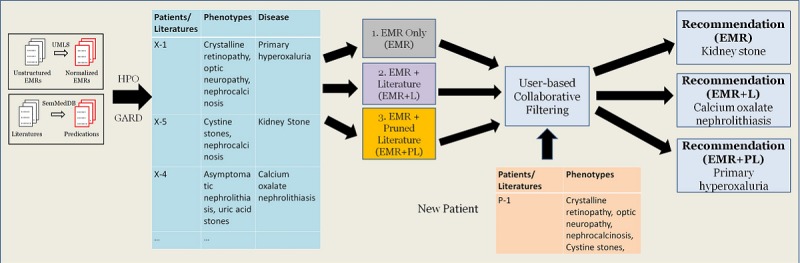
System workflow. EMR: electronic medical record; UMLS: Unified Medical Language System.

Figure 1 illustrates the system workflow of our study. The proposed system is able to absorb heterogeneous data sources, and it adapted the collaborative filtering model on any type of input for rare disease recommendation in a general manner. For EMRs, we leveraged the developed annotation pipeline to collect all phenotypic information mentioned within 1 year of the first appearance of the rare disease [4]. For medical literature, we first retrieved all predications from SemMedDB and saved them with PMID. We looked up HPO and GARD glossaries to check each predication (subject, predicate, object) and filtered out those sentences in which neither subject nor object could be found. To exclude disease-disease and phenotype-phenotype associations, we also filtered out those predications in which both the subject and the object could be mapped to the same vocabulary (GARD or HPO) and only kept the associations between phenotypes and rare diseases.

The format of input data is composed of patient identification or PMID and unique phenotypes manifested by each patient or article. We treated a positive diagnosis of a rare disease as a gold standard for association tasks involving patients and PMID to rare disease mentions as a gold standard for literature association tasks. We used 3 different data fusion strategies to prepare homogeneous and heterogeneous resources:

1. EMR only: Only patient-phenotype information extracted from the EMR was used.
2. EMR and literature (EMR+L): We first conducted a treatment on medical literature. Since each publication might only mention 1 phenotype with 1 rare disease, to strengthen the evidence power provided by the literature, we merged multiple literature sources together as 1 large document if those sources shared the same rare disease. Therefore, the number of documents used will be less than 91,680. We then mixed patient-phenotype association with literature-phenotype information and randomly permuted them without any additional treatment. Detailed steps of this process are shown as case 1 in Textbox 2.
3. EMR and pruned literature (EMR+PL). A similar approach as EMR+L was followed, but some phenotype-rare disease associations mined from literature were additionally filtered out if they did not appear in the EMR. In this case, we tried to enhance the correlation and coexisting evidence between phenotypes and rare diseases a bit further to provide a better prediction output. Case 2 in Textbox 2 demonstrates this pruned process.

Different phenotype-disease associations with 3 different data fusion strategies were imported to collaborative filtering model and the final recommendation outputs based on 3 data inputs would be given. For example, if a new patient has phenotypes *crystalline retinopathy*, *optic neuropathy*, *nephrocalcinosis*, and *cysteine stones*, 3 different disease recommendations (*kidney stone*, *calcium oxalate nephrolithiasis*, and *primary hyperoxaluria*) will be made, and we compared them with such patient’s true diagnostic results for evaluation purpose.

### Evaluation

We evaluated 24 various evaluation groups as: (1) TANI with KNN on EMR; (2) TANI with KNN on EMR and literature; (3) TANI with KNN on EMR and pruned literature; (4) TANI with TPN on EMR; (5) TANI with TPN on EMR and literature; (6) TANI with TPN on EMR and pruned literature; (7) LL with KNN on EMR; (8) LL with KNN on EMR and literature; (9) LL with KNN on EMR and pruned literature; (10) LL with TPN on EMR; (11) LL with TPN on EMR and literature; (12) LL with TPN on EMR and pruned literature; (13) OL with KNN on EMR; (14) OL with KNN on EMR and literature; (15) OL with KNN on EMR and pruned literature; (16) OL with TPN on EMR; (17) OL with TPN on EMR and literature; (18) OL with TPN on EMR and pruned literature; (19) FMG with KNN on EMR; (20) FMG with KNN on EMR and literature; (21) FMG with KNN on EMR and pruned literature; (22) FMG with TPN on EMR; (23) FMG with TPN on EMR and literature; and (24) FMG with TPN on EMR and pruned literature.

We used the same metrics adopted in our previous work to evaluate system performance. Specifically, we applied root mean square error (RMSE) [35] to determine the optimal thresholds for KNN and TPN. We evaluated performances of patient recommendations using precision-recall curve and mean average precision [5]. We also evaluated disease prediction performance with precision recall and *F*-measure [5].

Similar to our previous study, we used 3 matching strategies to measure the similarity between any 2 rare diseases: string matching, systematized nomenclature of medicine-clinical terms (SNOMED) matching, and GARD matching to provide different levels of relaxation on predicting rare diseases [5].

**Algorithm 2-Heterogeneous Data Fusion for electronic medical record and literature.**

Input: Map A (PMID, Rare Disease), Map B (PMID, Map(Rare Disease, List(Phenotypes)))

Output for Case 1: Merged literature with same rare disease, stored rare diseases along with their associated phenotypes in Map C

Output for Case 2: Pruned Map C’

Case 1: EMR+L

1. FOR each PMID and Rare Disease RD in A

2.      retrieve all relevant phenotypes {P} for RD and PMID from B

3.      IF C does not contain RD

4.         create new document_ID

5.         add {P} to list L

6.         add (document_ID, (RD, L)) to C

7.      ELSE

8.         List L=A.retrieve(document_ID)

9.         add nonduplicate elements from {P} to list L

10.       add (document_ID, (RD, L)) to C

11. RETURN C

Case 2: EMR+PL

12. C’=C

13. FOR each phenotype-disease pair PD1 in Map E

14.     FOR each phenotype-disease pair PD2 in Map C’

15.         IF (PD1 !=PD2)

16.         remove PD2 from C’

17. RETURN C’

## Results

As shown in Table 1, after eliminating rare diseases that affect only 1 patient, there were 38,607 patients for EMR only, 40,241 patients for EMR and literature, and 39,677 patients for EMR and pruned literature datasets. Since EMR+L is mixed data without any refinement, the total number of phenotypes, rare diseases, and their associations are larger than the other 2 outputs. In addition, of the 32 possible GARD categories, we found that the number of GARD categories covered for each of the 3 outputs were 28, 31, and 28, respectively.

### Threshold Selection With Root Mean Square Error

**Figure 2:**
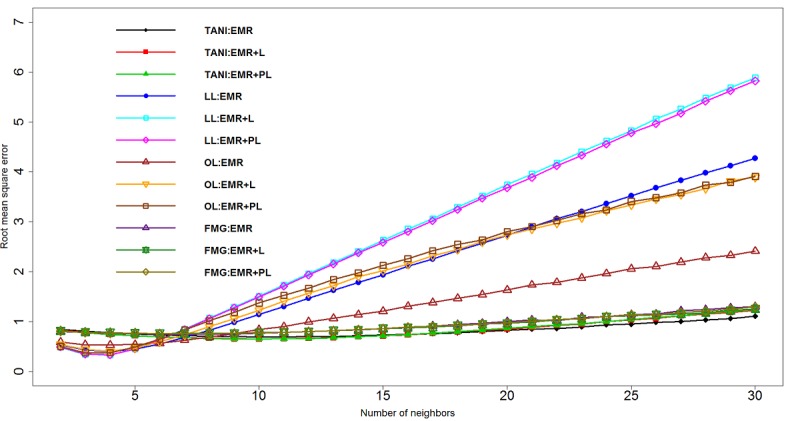
Root mean square error (RMSE) for k-nearest neighbors (KNN) with four similarity measurements. EMR: electronic medical record; FMG: Fager and McGowan coefficient similarity; L: literature; LL: log likelihood ratio similarity; OL: overlap coefficient similarity; PL: pruned literature; TANI: Tanimoto coefficient similarity.

For KNN combined with different similarity measurements, Figure 2 plots the curve to illustrate the change of RMSE associated with different number of selected neighbors. We observed that for LL and OL, RMSE was more sensitive to EMR+L and EMR+PL than to EMR only, which shows that adding sources of literature might affect the results in either a positive or negative way. On the other hand, the change of RMSE for TANI and FMG was minimal among these 3 datasets, which indicates that literature enrichment did not reflect markedly on the performance for these 2 algorithms.

**Table 1:** Statistics for prepared datasets.

Datasets	EMR^a^ only, n	EMR and literature (EMR+L), n	EMR and pruned literature (EMR+PL), n
Patients or literature sources	38,607	40,241	39,677
Phenotypes	3271	3818	3271
Rare diseases	1074	1634	1074
Phenotype-disease associations	141,036	154,802	141,036
GARD^b^ categories covered	28	31	28

^a^EMR: electronic medical record.

^b^GARD: Genetic and Rare Diseases Information Center.

**Figure 3:**
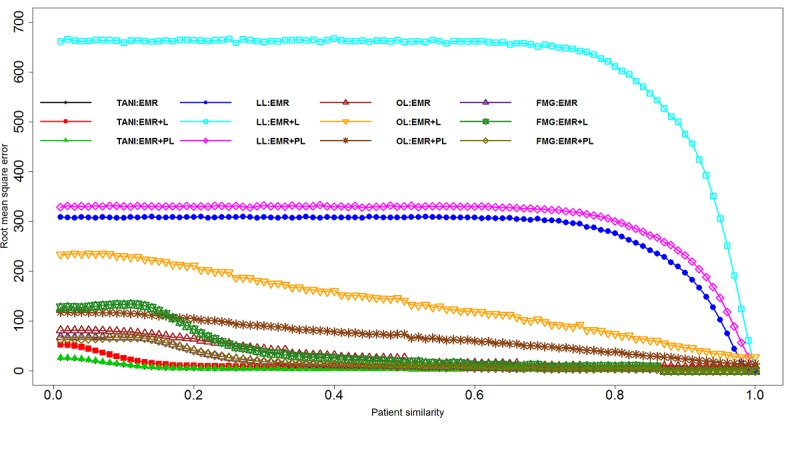
Root mean square error (RMSE) for threshold patient neighbor (TPN) with four similarity measurements. EMR: electronic medical record; FMG: Fager and McGowan coefficient similarity; L: literature; LL: log likelihood ratio similarity; OL: overlap coefficient similarity; PL: pruned literature; TANI: Tanimoto coefficient similarity.

Figure 3 describes how RMSE changes for the tested coefficient similarities with TPN. We found that TANI is sensitive to a smaller threshold but tends to be balanced with the threshold getting larger for all 3 datasets. RMSE for LL remained balanced until similarity threshold became larger, denoting that LL is not sensitive to the similarity threshold. All 4 algorithms held a higher average RMSE with EMR+L but a lower average RMSE with EMR and EMR+PL, indicating that the random mix of EMR and literature might not be able to provide a strong scheme for rare disease prediction and recommendation. Specifically for OL, EMR only performed better than the other 2 datasets, which showed that OL is not a very suitable measurement for heterogeneous datasets. Table 2 summarizes the optimal threshold selection for different evaluation groups.

### Performance for Patient Recommendation

**Figure 4:**
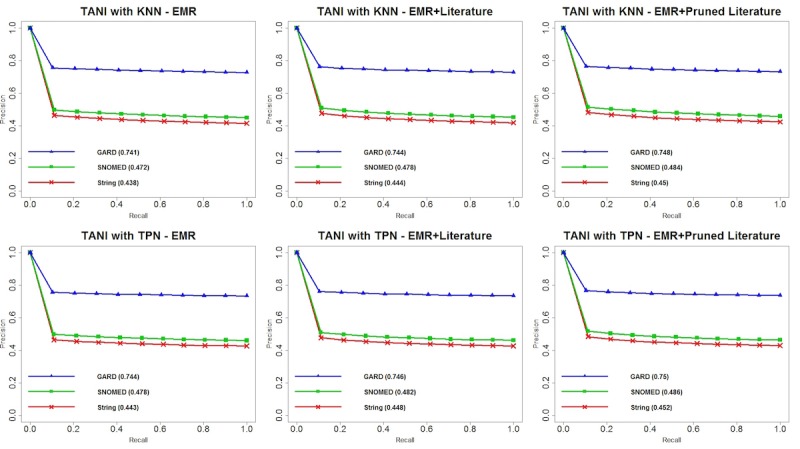
Precision-recall curves and area under the precision-recall curve (PRAUC) for Tanimoto coefficient similarity (TANI) with k-nearest neighbors (KNN) and threshold patient neighbors (TPN). EMR: electronic medical record; GARD: Genetic and Rare Diseases Information Center; KNN: k-nearest neighbors; SNOMED: systematized nomenclature of medicine; TANI: Tanimoto coefficient similarity.

**Figure 5:**
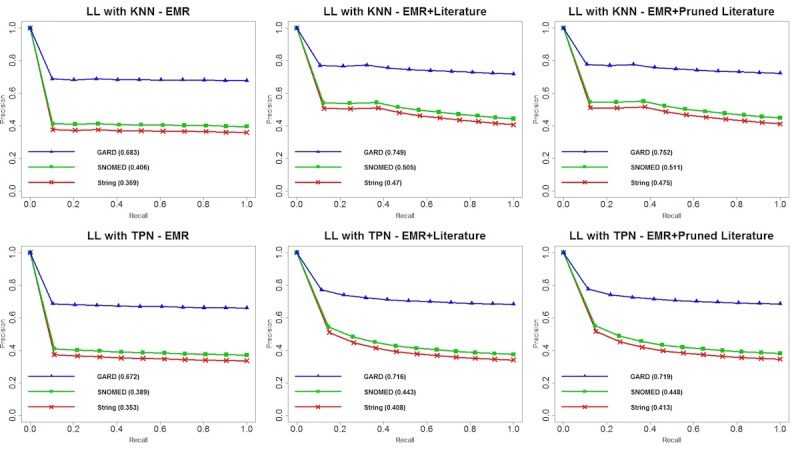
Precision-recall curves and area under the precision-recall curve (PRAUC) for log likelihood ratio similarity with k-nearest neighbors and threshold patient neighbors. EMR: electronic medical record; GARD: Genetic and Rare Diseases Information Center; KNN: k-nearest neighbors; LL: log likelihood ratio similarity; SNOMED: systematized nomenclature of medicine; TPN: threshold patient neighbor.

**Figure 6:**
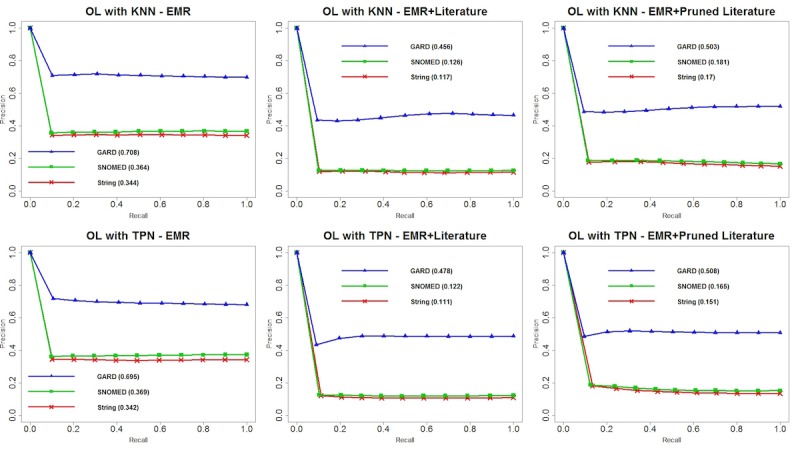
Precision-recall curves and area under the precision-recall curve (PRAUC) for overlap coefficient similarity with k-nearest neighbors and threshold patient neighbors. EMR: electronic medical record; GARD: Genetic and Rare Diseases Information Center; KNN: k-nearest neighbors; OL: overlap coefficient similarity; SNOMED: systematized nomenclature of medicine; TPN: threshold patient neighbor.

**Figure 7:**
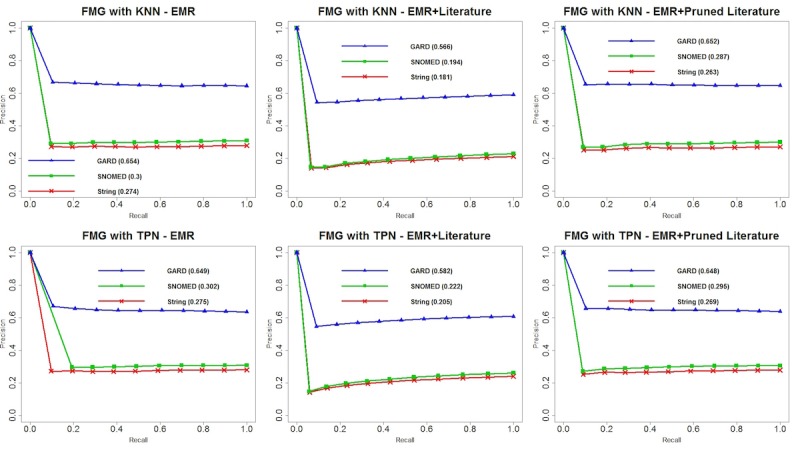
Precision-recall curves and area under the precision-recall curve (PRAUC) for Fager and McGowan coefficient similarity with k-nearest neighbors and threshold patient neighbors. EMR: electronic medical record; FMG: Fager and McGowan coefficient similarity; GARD: Genetic and Rare Diseases Information Center; KNN: k-nearest neighbors; SNOMED: systematized nomenclature of medicine; TPN: threshold patient neighbor.

We plotted precision-recall curves for each of the 24 experiments and area under the precision-recall curve (PRAUC) for each matching criterion. Overall, we observed that GARD matching contributed to the optimal performance among all matching criteria, and SNOMED semantic matching was always a suboptimal strategy. Figure 4 shows the performance of TANI with KNN and TPN on different datasets and matching criteria. We observed that there are no considerable differences between TANI+KNN and TANI+TPN for 3 matching criteria with 3 datasets. Although the difference seems subtle, TANI+TPN with EMR+PL yielded the optimal PRAUC score for string, SNOMED, and GARD matching, respectively. Figure 5 shows the performance of LL. Compared with TANI, LL performed worse with TPN for all datasets and matching criteria. However, when using KNN, although LL performed worse with EMR data only, it outperformed for both EMR+L and EMR+PL. This result indicates that LL is more suitable for mining knowledge from heterogeneous datasets than TANI. Figure 6 illustrates the performance of OL. Compared with TANI and LL, this measurement produced considerably lower PRAUC for either neighborhood algorithm. Additionally, OL yielded better performance with EMR data only but worse performance with combined datasets, which indicates that OL may be more suitable for a single dataset, and it suggests that combined datasets might possess too much noise for OL to make an accurate judgment. Although OL cannot handle literature-enriched data well, we observed that pruned literature still performed better than nonpruned literature. Figure 7 depicts the reaction of FMG to different neighborhood algorithms and combinations of datasets. Similar to OL, FMG with EMR data only outperformed EMR+L and EMR+PL in all 3 matching criteria. However, unlike OL, although FMG with EMR+L had the worst performance with both KNN and TPN, pruned literature slightly increased the performance, and no substantial difference exists between using FMG with EMR only and EMR+PL. Tables 3-6 show MAP for all patients’ recommendations, which showed a consistent performance with PRAUC evaluation, indicating that TANI and LL performed better and are more suitable for integrated EMR and literature, whereas OL and FMG performed worse and are not suitable for fused datasets. In general, optimal performance produced by LL indicated the potential of combining EMR and literature to increase patient stratification.

**Table 2:** Optimal thresholds for different evaluation groups.

Optimal parameters	TANI^a^			LL^b^			OL^c^			FMG^d^		
	EMR^e^	EMR+L^f^	EMR+PL^g^	EMR	EMR+L	EMR+PL	EMR	EMR+L	EMR+PL	EMR	EMR+L	EMR+PL
Optimal *k* (KNN^h^)	11	10	9	4	4	4	4	4	4	7	6	6
Optimal *t* (TPN^i^)	0.19	0.19	0.2	0.72	0.73	0.76	0.51	0.49	0.51	0.12	0.11	0.12

^a^TANI: Tanimoto coefficient similarity.

^b^LL: log likelihood ratio similarity.

^c^OL: overlap coefficient similarity.

^d^FMG: Fager and McGowan coefficient similarity.

^e^EMR: electronic medical record.

^f^L: literature.

^g^PL: pruned literature.

^h^KNN: k-nearest neighbors.

^i^TPN: threshold patient neighbor.

**Table 3:** Mean average precision for TANI^a^ with EMR^b^, EMR+L^c^, and EMR+PL^d^ (optimal in italics).

Matching criterion	EMR		EMR+L		EMR+PL	
	KNN^e^	TPN^f^	KNN	TPN	KNN	TPN
String	0.435	0.441	0.436	0.445	*0.446*	*0.448*
SNOMED^g^	0.469	0.475	0.474	0.479	*0.481*	*0.483*
GARD^h^	0.739	0.742	0.742	0.745	*0.746*	*0.748*

^a^TANI: Tanimoto coefficient similarity.

^b^EMR: electronic medical record.

^c^L: literature.

^d^PL: pruned literature.

^e^KNN: k-nearest neighbors.

^f^TPN: threshold patient neighbor.

^g^SNOMED: systematized nomenclature of medicine.

^h^GARD: Genetic and Rare Diseases Information Center.

**Table 4:** Mean average precision for LL^a^ with EMR^b^, EMR+L^c^, and EMR+PL^d^ (optimal in italics).

Matching criterion	EMR		EMR+L		EMR+PL	
	KNN^e^	TPN^f^	KNN	TPN	KNN	TPN
String	0.368	0.351	0.46	0.391	*0.465*	*0.396*
SNOMED^g^	0.405	0.386	0.495	0.426	*0.5*	*0.431*
GARD^h^	0.683	0.67	0.745	0.71	*0.749*	*0.713*

^a^LL: log likelihood ratio similarity.

^b^EMR: electronic medical record.

^c^L: literature.

^d^PL: pruned literature.

^e^KNN: k-nearest neighbors.

^f^TPN: threshold patient neighbor.

^g^SNOMED: systematized nomenclature of medicine.

^h^GARD: Genetic and Rare Diseases Information Center.

**Table 5:** Mean average precision for OL^a^ with EMR^b^, EMR+L^c^, and EMR+PL^d^ (optimal in italics).

Matching criterion	EMR		EMR+L		EMR+PL	
	KNN^e^	TPN^f^	KNN	TPN	KNN	TPN
String	*0.344*	*0.342*	0.117	0.11	0.167	0.148
SNOMED^g^	*0.365*	*0.369*	0.126	0.122	0.179	0.162
GARD^h^	*0.708*	*0.693*	0.457	0.48	0.505	0.509

^a^OL: overlap coefficient similarity.

^b^EMR: electronic medical record.

^c^L: literature.

^d^PL: pruned literature.

^e^KNN: k-nearest neighbors.

^f^TPN: threshold patient neighbor.

^g^SNOMED: systematized nomenclature of medicine.

^h^GARD: Genetic and Rare Diseases Information Center.

**Table 6:** Mean average precision for FMG^a^ with EMR^b^, EMR+L^c^, and EMR+PL^d^ (optimal in italics).

Matching criterion	EMR		EMR+L		EMR+PL	
	KNN^e^	TPN^f^	KNN	TPN	KNN	TPN
String	*0.274*	*0.275*	0.18	0.205	0.264	0.27
SNOMED^g^	*0.3*	*0.302*	0.192	0.221	0.288	0.297
GARD^h^	*0.653*	*0.647*	0.568	0.584	0.651	*0.647*

^a^FMG: Fager and McGowan coefficient similarity.

^b^EMR: electronic medical record.

^c^L: literature.

^d^PL: pruned literature.

^e^KNN: k-nearest neighbors.

^f^TPN: threshold patient neighbor.

^g^SNOMED: systematized nomenclature of medicine.

^h^GARD: Genetic and Rare Diseases Information Center.

### Performance on Rare Disease Prediction With Log Likelihood Ratio Similarity

**Figure 8:**
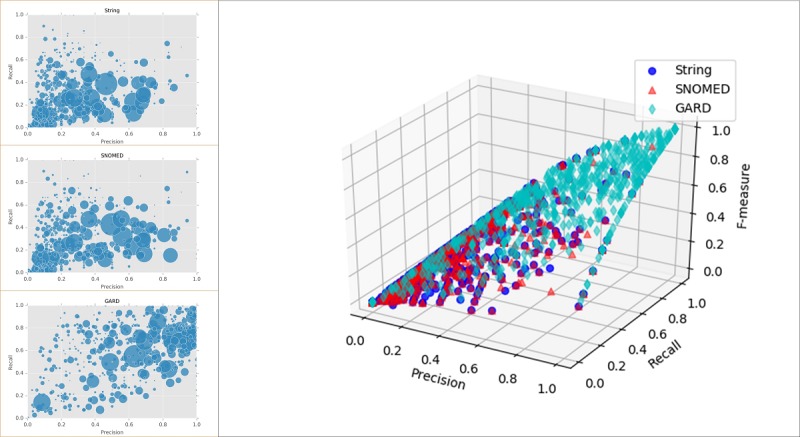
Prediction performance for rare diseases. GARD: Genetic and Rare Diseases Information Center; SNOMED: systematized nomenclature of medicine.

We selected LL with KNN as the optimal metric, trained it with EMR+PL, and applied it on 44,060 patients with only 1 rare disease. We only selected rare diseases with at least 3 affected patients, which resulted in 702 rare diseases in total. Prediction performances for different matching criteria are described as shown in Figure 8. The circle size in two-dimensional scatter plots is proportional to the number of affected patients. Three-dimensional plot for precision, recall, and *F*-measure in Figure 8 clearly depicts that GARD outperformed SNOMED matching, and string matching yielded the worst performance. Macro-average *F*-measure for string, SNOMED, and GARD matching for the tested diseases were 0.32, 0.42, and 0.63, respectively.

In Table 7, we selected 9 diseases for each matching criterion for LL with KNN. Specifically, we picked 3 with high *F*-measures, 3 with medium to high *F*-measures, and 3 with relatively low *F*-measures. For any rare disease affecting no more than 10 cases, we marked them as <10.

For string matching, *holoprosencephaly*, *Huntington disease*, and *juvenile polyposis syndrome* contributed to higher *F*-measures and do not have a large number of affected patients. However, since they are unique, performance of recommendation was promising. *Sacrococcygeal teratoma*, *frontotemporal dementia*, and *polycystic liver disease* were well predicted but with some missed cases. Taking *sacrococcygeal teratoma* as an example, we found *neurogenic bladder*, *constipation*, and *diarrhea* to be the most common phenotypes that also occurred in patients with the rare disease *microcephaly*.

**Table 7:** Recommendation performance for selected rare diseases (3 high, 3 medium to high, 3 low).

Approaches and top diseases		Number of patients affected	Precision	Recall	*F*-measure
**LL^a^+KNN^b^ with string matching**					
	Holoprosencephaly	<10	0.75	1	0.86
	Huntington disease	<10	1	0.67	0.8
	Juvenile polyposis syndrome	<10	0.91	0.71	0.8
	Sacrococcygeal teratoma	15	0.83	0.67	0.74
	Frontotemporal dementia	202	0.69	0.58	0.63
	Polycystic liver disease	72	0.64	0.58	0.61
	Hemicrania continua	36	0.08	0.25	0.12
	Intrahepatic cholangiocarcinoma	94	0.08	0.22	0.12
	Neuromyelitis optica	50	0.16	0.1	0.12
**LL+KNN with SNOMED^c^ matching**					
	Myxoid liposarcoma	37	0.94	0.89	0.91
	Linear scleroderma	16	0.91	0.71	0.8
	Migraine with brainstem aura	15	0.75	1	0.86
	Hypophosphatemic rickets	<10	0.83	0.75	0.79
	Congenital radio ulnar synostosis	14	0.67	0.86	0.75
	Spasmodic dysphonia	177	0.83	0.67	0.74
	Acute graft-versus-host disease	20	0.1	0.5	0.15
	Cryptogenic organizing pneumonia	37	0.14	0.17	0.15
	Cerebellar degeneration	29	0.14	0.17	0.15
**LL+KNN with GARD^d^ matching**					
	Acrospiroma	<10	1	1	1
	Birt-Hogg-Dube syndrome	<10	1	1	1
	Dendritic cell tumor	<10	1	1	1
	Acute promyelocytic leukemia	15	0.97	0.93	0.95
	Migraine with brainstem aura	15	1	0.88	0.93
	Thyroid cancer, anaplastic	30	1	0.86	0.92
	Addison disease	34	0.88	0.45	0.6
	Encephalocele	56	0.4	0.59	0.48
	Mixed connective tissue disease	78	0.4	0.48	0.43

^a^LL: log likelihood ratio similarity.

^b^KNN: k-nearest neighbors.

^c^SNOMED: Systematized Nomenclature of Medicine.

^d^GARD: Genetic and Rare Diseases Information Center.

In our EMR, *sacrococcygeal teratoma* patients and *microcephaly* patients reported 140 cases of *neurogenic bladder*, 84 cases of *constipation*, and 84 cases of *diarrhea*. In medical literature, *neurogenic bladder*, *constipation*, and *diarrhea* are also the 3 top phenotypes found in microcephaly, and they appeared 34, 32, and 32 times, respectively. Considering evidence from heterogeneous data sources, *sacrococcygeal teratoma* was often predicted as *microcephaly*. *Hemicrania continua*, *intrahepatic cholangiocarcinoma*, and *neuromyelitis optica* are 3 diseases with relatively low *F*-measures. Although the number of affected patients for them is not small, they lack a unique group of phenotypic patterns to differentiate them from other diseases with similar phenotypes.

For SNOMED matching, the top predicted diseases are *myxoid liposarcoma*, *linear scleroderma*, and *microscopic polyangiitis*. Since we used the SNOMED semantic hierarchy to measure the similarity between 2 diseases, the prediction performance was slightly better than using string matching only. For example, in our results, *myxoid liposarcoma* was semantically the same as *liposarcoma*, *linear scleroderma* had the same meaning as *morphea*, and *microscopic polyangiitis* was treated equally with *granulomatosis with polyangitis*. These 3 diseases are related to unique phenotypes, and as such, the prediction results were positive. For example, the phenotypes *soft tissue sarcoma* and *lymphedema* have a tight relationship with *myxoid liposarcoma*. In addition, 65 phenotypes from EMRs and literature were closely related to *linear scleroderma*, and we found that *headache* and *hemiatrophy* frequently appeared. We also found that *vasculitis* and *glomerulonephritis* often appeared along with *cicatricial pemphigoid*. *Hypophosphatemic rickets*, *congenital radio ulnar synostosis*, and *spasmodic dysphonia* also contributed to the positive recommendation results but with some minor prediction errors. For example, *hypophosphatemic rickets* was considered to be *lung adenocarcinoma* in a few cases, *congenital radio ulnar synostosis* was misdiagnosed as *esophageal atresia*, and *spasmodic dysphonia* and *trigeminal neuralgia* were sometimes mismatched. Finally, patient profiles from EMRs and literature content regarding *acute graft-versus-host disease*, *cryptogenic organizing pneumonia*, and *cerebellar degeneration* were not good enough for our model to conduct the prediction.

Since GARD matching was able to have a broader recommendation based on system categories of rare diseases, it usually yielded a better prediction performance than the other 2 strategies. *Acrospiroma*, *Birt-Hogg-Dube syndrome*, and *dendritic cell tumor* all had a 100% prediction rate, though the number of affected patients was small. By using GARD matching, for example, *acrospiroma* can be inferred as *fibrosarcoma*, *Birt-Hogg-Dube syndrome* can be recommended as syndrome *adenocarcinoma of the appendix*, and *dendritic cell tumor* can be predicted as *large granular lymphocyte leukemia*. The reason for this is that all of these pairs can be categorized as rare cancers according to GARD. Similarly, in *acute promyelocytic leukemia*, *migraine with brainstem aura*, and *thyroid cancer*, *anaplastic disease* can also be recommended to other diseases within the same rare disease system. For some rare diseases, the GARD matching did not perform well. In the case of *Addison disease*, for example, although we found some recommendations by GARD matching from our datasets, such as *isolated ACTH deficiency* (categorized in Endocrine Diseases), *x-linked adrenal hypoplasia congenital* (categorized in Congenital and Genetic Diseases), *fibrous dysplasia* (categorized in Congenital and Genetic Diseases), and *syringomyelia* (categorized in Congenital and Genetic Diseases), there are still many nonrelevant results found by our system caused by general phenotypes that are related to numerous diseases. In general, the prediction of similar kind of rare diseases can still provide suggestions and clues for physicians’ decision making.

## Discussion

### Limitations

This study demonstrates the potential to provide decision support on rare diseases for differential diagnosis. With more comprehensive knowledge extracted from clinical notes and literature, collaborative filtering performed better on both patient recommendation and rare disease prediction. The current clinical decision support (CDS) system is limited to a narrow area of clinical practice due to the inability to utilize information embedded in clinical narratives and challenges in making good semantic alignment among precision medicine knowledge and clinical data stored in various formats and heterogeneous resources. Therefore, there exists a huge opportunity to integrate our proposed work into current CDS system for a better rare disease differential diagnosis in clinical practice.

For homogeneous data, LL performance would be depressed when compared with TANI (eg, EMR only). On the other hand, LL is good at dealing with heterogeneous data, and as phenotype-rare disease associations extracted from EMR and medical literature share different perspectives, such flexibility can help us find more patterns compared with TANI. Therefore, it is not surprising that patient recommendation performance improved when we combined EMR and literature randomly, and performance improved further after we used pruned literature. OL and FMG, however, performed worse than TANI and LL. We found that OL gives too much weight to patient similarities even with few shared phenotypes. Such strict similarity measurements have difficulty finding semantic relationships and lack the ability to stratify patients well. This is possibly an explanation for the better performance of OL for single EMR data with high homogeneity but poor performance for combined datasets with high heterogeneity. Similar to OL, FMG is not good at dealing with heterogeneous data; nevertheless, it yielded a better patient recommendation performance than OL in the EMR+L and EMR+PL datasets. Furthermore, we observed that LL is sensitive to the selection of KNN or TPN, especially for combined datasets, which infers that making a good balance between KNN and TPN has the potential ability to optimize overall performance and eliminate bias with idealized neighbors and similarity at the same time.

The combination of EMR and literature did not always contribute to optimal performance in patient recommendations. The reason for this is that some biases exist when physicians or researchers documented phenotype-disease associations. For EMRs, each document is recorded based on individual physician instinct and experiences starting from a clinical perspective, and for literature, phenotypes and rare diseases with positive relationships are reported based on a large number of gene tests from a biomedical experimental perspective, which may increase the gap between these two sources. Collaborative filtering with different similarity measurements and neighborhood algorithms can remedy this problem to some extent. In the future, we plan to investigate on gene level to reduce miscommunication and balance the heterogeneity between different datasets. Besides the use of literature only, it would also be interesting to integrate cross-institutional EMRs with balanced heterogeneity to acquire diagnostic experience and knowledge from multiple hospitals and health care institutions to build a more general system for rare disease diagnostic decision support.

### Conclusion and Future Work

We investigated the application of a patient-based collaborative filtering model on heterogeneous EMRs and literature with different similarity measurements and neighborhood algorithms. Results demonstrated the potential of combining heterogeneous datasets to support diagnostic decision making for rare diseases.

In the future, we are going to fully utilize the graph structure provided by the HPO and leverage its node embeddings [5,36,37] to provide coefficient similarities from various perspectives to improve performance of disease recommendation. We also plan to resolve the heterogeneity issue and reduce miscommunication between EMR and literature by mining genotypic information to establish a comprehensive disease-phenotype-gene network for rare disease diagnosis.

## Abbreviations

CDS: clinical decision support
EMR: electronic medical record
FMG: Fager and McGowan coefficient similarity
GARD: Genetic and Rare Diseases Information Center
HPO: Human Phenotype Ontology
KNN: K-nearest neighbors
LL: log likelihood ratio similarity
OL: overlap coefficient similarity
PRAUC: area under the precision-recall curve
RMSE: root mean square error
SNOMED: systematized nomenclature of medicine
TANI: Tanimoto coefficient similarity
TPN: threshold patient neighbor
